# Shock Index as a Predictor for Angiographic Hemostasis in Life-Threatening Traumatic Oronasal Bleeding

**DOI:** 10.3390/ijerph182111051

**Published:** 2021-10-21

**Authors:** Fang-Yu Hsu, Shih-Hsuan Mao, Andy Deng-Chi Chuang, Yon-Cheong Wong, Chih-Hao Chen

**Affiliations:** 1 Craniofacial Research Center and Department of Plastic and Reconstructive Surgery, Chang Gung Memorial Hospital at Linkou, College of Medicine, Chang Gung University, Taoyuan 333, Taiwan; sky9832@hotmail.com (F.-Y.H.); raymao0304@gmail.com (S.-H.M.); 2 Department of Plastic and Reconstructive Surgery, Chang Gung Memorial Hospital at Keelung, Keelung 204, Taiwan; andy.d.chuang@gmail.com; 3 Department of Diagnostic Radiology, Chang Gung Memorial Hospital at Linkou, College of Medicine, Chang Gung University, Taoyuan 333, Taiwan; ycwong@cgmh.org.tw

**Keywords:** traumatic oronasal bleeding, transarterial embolization, craniofacial trauma, shock index, systolic blood pressure, heart rate

## Abstract

The objective of this retrospective study was to identify predictors of angiographic hemostasis among patients with life-threatening traumatic oronasal bleeding (ONB) and determine the threshold for timely referral or intervention. The diagnosis of traumatic, life-threatening ONB was made if the patient suffered from craniofacial trauma presenting at triage with unstable hemodynamics or required a definitive airway due to ONB, without other major bleeding identified. There were 4404 craniofacial trauma patients between January 2015 and December 2019, of which 72 (1.6%) fulfilled the diagnosis of traumatic life-threatening ONB. Of these patients, 39 (54.2%) received trans-arterial embolization (TAE), 11 (15.3%) were treated with other methods, and 22 (30.5%) were excluded. Motor vehicle accidents were the most common cause of life-threatening ONB (52%), and the internal maxillary artery was the most commonly identified hemorrhaging artery requiring embolization (84%). Shock index (SI) was significantly higher in the angiographic hemostasis group (*p* < 0.001). The AUC-ROC was 0.87 (95% CI, 0.88–1.00) for SI to predict angiographic hemostasis. Early recognition and timely intervention are crucial in post-traumatic, life-threatening ONB management. Patients initially presenting with SI > 0.95 were more likely to receive TAE, with the TAE group having statistically higher SI than the non-TAE group whilst receiving significantly more packed red blood cells. Hence, for patients presenting with life-threatening traumatic ONB and a SI > 0.95, TAE should be considered if preliminary attempts at hemostasis have failed.

## 1. Introduction

Life-threatening oronasal bleeding (ONB) after craniofacial trauma is infrequent with an approximate incidence rate of 1%, but mortality rates can be as high as 85.9% if hemostasis is not achieved [1,2,3,4,5]. High rates of mortality in traumatic, life-threatening ONB remain a challenge for clinical physicians [1,3,6,7]. Timely and adequate intervention is critical in reducing hemorrhage-related mortality, while delayed recognition and subsequent management may lead to unfavorable outcomes and dire consequences [1,3,8,9,10]. For patients presenting with traumatic ONB, clinical management follows Advanced Trauma Life Support (ATLS) protocols along with airway protection, vital sign stabilization, bleeder identification, and hemostasis. The current consensus is to perform conservative treatment first (consisting of fluid resuscitation, blood transfusion, and anterior and posterior nasal packing) followed by trans-arterial embolization (TAE). However, the association of TAE with several major complications such as cerebrovascular accident, nephrotoxicity, facial nerve palsy, monocular blindness, and soft tissue necrosis have been previously reported in the literature [11,12,13]. Furthermore, TAE requires adequately trained interventional radiologists with proper facilities and equipment, both of which may not be readily available at all institutions. Based on the availability of trained personnel and facilities, treatment may potentially be delayed until the proper staff and facilities are secured. As a delay in intervention increases the complication rate, prompt recognition of patients requiring TAE is crucial. This study aims to identify possible predictors for patients requiring angiographic hemostasis, allowing first-line physicians to respond accordingly.

## 2. Materials and Methods

**Figure 1:**
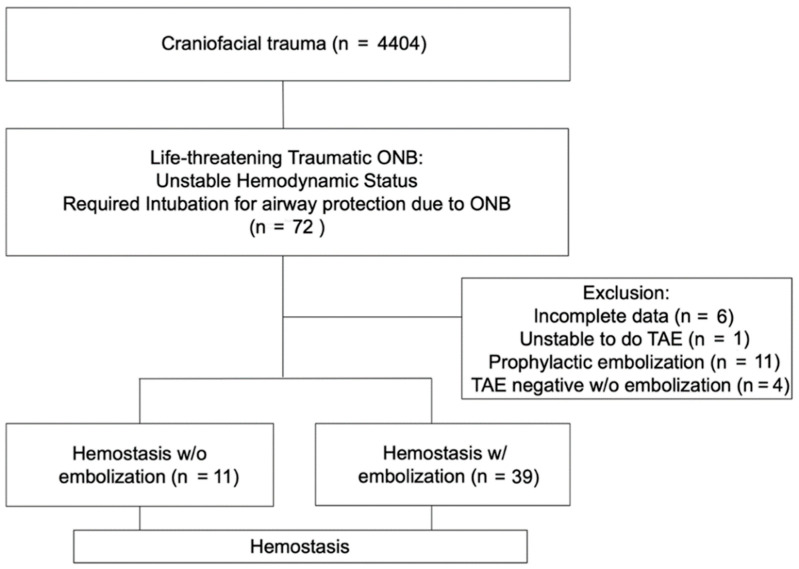
The flowchart of patients who met inclusion/exclusion criteria of this study.

This study was conducted retrospectively and approved by the Institutional Review Board (#104-2789B). Patients with craniofacial trauma were recruited in a tertiary referral center from January 2015 to December 2019 (Figure 1). All patients were managed according to ATLS protocol. Diagnosis of life-threatening ONB was made if the patient was bleeding from the oronasal area with accompanying unstable hemodynamics, had systolic blood pressure (SBP) < 90 mmHg or heart rate (HR) > 100 beats per minute, or required a definitive airway establishment due to excessive hemorrhage. Patients were excluded if they died before TAE, underwent angiography without embolization, received embolization without evidence of contrast extravasation or pseudoaneurysm during angiography, or had incomplete medical records. Patients were included if they were managed by conservative treatment, consisting of fluid resuscitation, blood transfusion, anterior nasal packing with a gauze, and/or posterior nasal packing with 10–14 Fr Foley catheters with 10–30 mL balloons. TAE was indicated if there was persistent uncontrolled bleeding or unstable hemodynamics despite conservative management as assessed by the physician on site. Evidence of active contrast extravasation or pseudoaneurysm during angiography was also considered a positive finding and mandated embolization. All patients included in this study must have reached transient stability after initial management at the emergency department and did not experience persistent hemorrhage.

We compared two group of patients. The first group consisted of patients who did not require TAE for hemostasis (non-TAE group) and the second group consisted of patients requiring TAE for hemostasis (TAE group). The medical records were independently collected by two physicians. The radiological images, findings, and procedures were reviewed by an experienced radiologist. Patient characteristics, vital signs, laboratory data, mechanism of injury, associated injury, patterns of facial fractures, amount of blood transfusion, TAE procedures and findings (if applicable), and clinical outcome were recorded. The Shock Index (SI) for each patient was also calculated from the vital signs measured after completion of anterior and posterior nasal packing (SI = HR/SBP). Data was compiled and analyzed using the SPSS package Version 20.0 for Windows. The data was summarized with nominal variables expressed with counts and percentages, while continuous variables were presented as mean ± SD. Variables were analyzed using Fisher’s exact test and Wilcoxon signed-rank test for nominal and continuous variables, respectively. The level of significance was set at *p <* 0.05. Receiver operating characteristic (ROC) curve was used to evaluate the performance of variables and to determine the cut-off value.

## 3. Results

**Figure 2:**
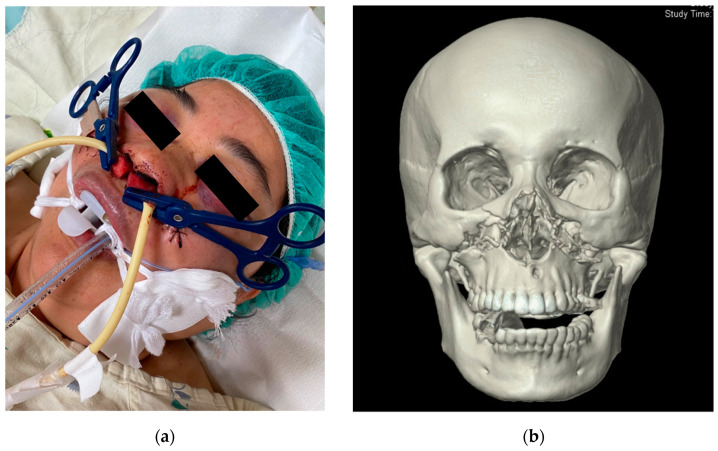
A 55-year-old female who presented with massive oronasal bleeding after facial trauma. (**a**) Patient was intubated for airway protection. Arterial and posterior nasal packing was performed after intubation. (**b**) CT scan shows bilateral maxillary fractures.

**Figure 3:**
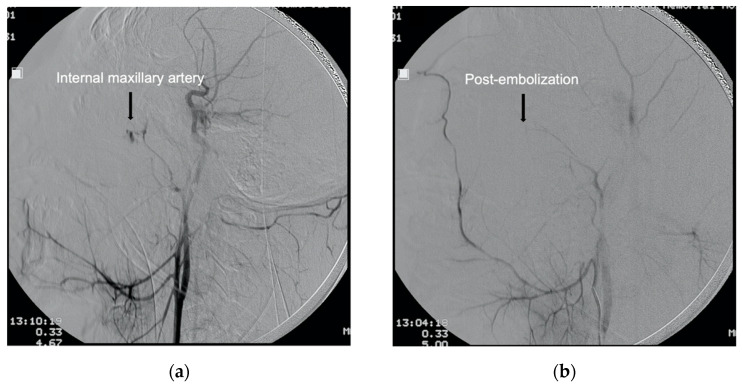
Angiogram of the patient shown in Figure 2. (**a**) Lateral angiogram of common carotid artery shows contrast extravasation from internal maxillary artery (arrow). (**b**) Post gelatin foam embolization images showing hemostasis (arrow).

There were 4404 craniofacial trauma patients with 72 fulfilling the criteria of life-threatening traumatic ONB (1.6%). Twenty-two patients were excluded due to incomplete data (*n* = 6), having undergone TAE without indication for angioembolization (*n* = 11), expiring before TAE could be performed (*n* = 1), and negative findings on angiography (*n* = 4) (Figure 1). The remaining fifty patients were included in this study. Motor vehicle accidents were the most common cause of life-threatening ONB (52%) with motorcycle accidents accounting for 68% of them. Patients were predominantly male (76%), in their 30s (age 35.1 ± 18.8), and suffered from blunt trauma to the facial region (98%). Eleven patients achieved hemostasis after nasal packing, while the other 39 patients only achieved hemostasis after TAE. All patients who required TAE for hemostasis were confirmed to have contrast extravasation or pseudoaneurysm formation of the external carotid artery origin, with the internal maxillary artery being the most commonly identified hemorrhaging artery (84%) (Figure 2 and Figure 3).

**Figure 4:**
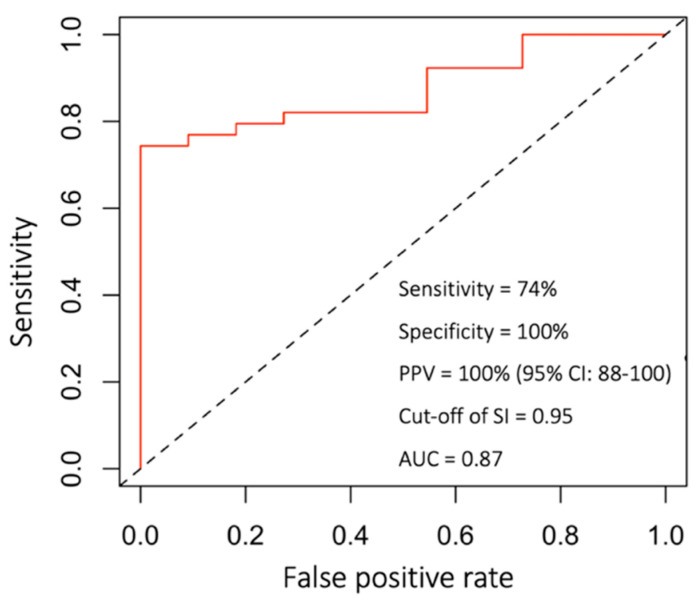
ROC curve of shock index and prediction of angioembolization. Cutoff value of SI, 0.95; sensitivity, 74%; specificity, 100%; PPV, 100%.

Fracture patterns, mechanism of injury, associated injuries, GCS, and hematocrit did not reach statistical difference when comparing the two groups. SBP at triage in the TAE group was significantly lower than in the non-TAE group (*p* = 0.01). HR at triage in the TAE group was significantly higher than in the non-TAE group (*p* = 0.004) (Table 1). Hence, the average SI at presentation for the TAE group (1.31 ± 0.49) was much higher than that calculated for the non-TAE group (0.73 ± 0.17) (*p* < 0.05). After nasal packing, SI for the TAE group improved (1.14 ± 0.37) compared to baseline values, but the change was not statistically significant. SI for the non-TAE group did not change (0.74 ± 0.23). SI for the TAE group was assessed after TAE (0.9 ± 0.31) and showed a significant improvement from baseline values (*p* < 0.05). The amount of blood transfused with packed red blood cells (PRBC) at the emergency department was 6.61 ± 5.0 units in the TAE group and 2.9 ± 3.2 units in the non-TAE group. The AUC-ROC curve was 0.87 (95%, CI = 0.88–1.00) for SI > 0.95 to predict successful angiographic hemostasis (Figure 4).

One patient had a palatal wound infection and required a local flap for reconstruction. Two patients had nasal-oral fistula formation requiring surgical intervention. Eight patients died of traumatic brain injury (TBI). Two patients died of delayed hemorrhage of non-oronasal origin, with one incidence of retroperitoneal hemorrhage and one incidence of pulmonary hemorrhage. No mortality was directly linked to ONB.

Seven patients experienced rebleeding events after TAE, and three of these required secondary interventions to reach hemostasis (Table 2). Six out of the seven rebleeding events occurred within 3 days after initial TAE. There were no sequelae or major complications associated with angiography.

## 4. Discussion

The incidence of life-threatening ONB after craniofacial trauma was reported to be about 1% [1,2,3,4,5]. In our study, the incidence of life-threatening ONB after craniofacial trauma was 1.6%. In these patients with severe, life-threatening ONB, those who required intervention with TAE had significantly higher SI at presentation (1.14 ± 0.37) compared to those who did not require further intervention (0.74 ± 0.23). Although ONB is diagnosed clinically, delayed management could occur from underestimating the severity and amount of blood loss, which may lead to unfavorable outcomes. ONB should be regarded as a consequential source of bleeding in the presence of hypovolemic shock in major trauma patients despite its relatively low incidence, as mortality rate and development of serious complications positively correlate to delay in management [1,3,9]. Early recognition, timely referral, and prompt intervention are imperative in approaching ONB patients. To that end, according to our findings, shock index may be a promising early predictor of severe disease in oronasal trauma patients, as the parameters required for calculating shock index are readily available at triage. In terms of management, TAE has been proven to be an effective method in hemostasis and has replaced surgery as the first line invasive procedure in ONB [1,14,15,16,17,18]. In this study, TAE played a role in hemodynamical stabilization in ONB with SI having significantly improved after TAE. TAE is safe, more selective, less invasive, and allows access to bleeding sites otherwise inaccessible to surgery. Despite its obvious advantages over more conservative treatment, TAE requires both specially trained radiologists and appropriate equipment. Such resources may not be readily available in every hospital, especially small-scale primary medical institutions. This limitation accentuates the importance of recognizing the need of TAE in the context of ONB. To the best of our knowledge, no parameter has been identified to be an effective predictor for the necessity of TAE in ONB. Hence, in order to help primary institutions, where resources for TAE may not be available, provide the patient with timely referral and arrange appropriate intervention as needed, this retrospective study was designed, which identifies possible predictors for the necessity of TAE in life-threatening traumatic ONB.

Motor vehicle accidents are the main cause of life-threatening ONB worldwide [3,14]. The concept of “golden time” emphasizes the importance of urgent care in the management of trauma patients. Effective field triage is essential in reducing transport time. If the injury exceeds the capability of the hospital providing first-aid, patients should be transferred to a higher-level facility promptly, as the availability of care at a trauma center is shown to directly affect patient mortality [19]. In the context of life-threatening ONB, prudent decision-making for referral to another institution may be a matter of life or death because interventional radiologists may not be readily available at every medical facility. 

**Figure 5:**
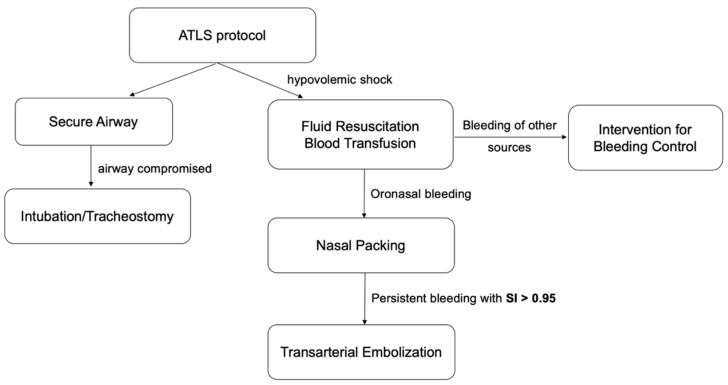
An algorithm for the management of life-threatening oronasal bleeding after craniofacial trauma.

Vital signs, heart rate, respiratory rate, blood pressure, and body temperature are fundamental measurements and are easily accessible by emergency medical responders (EMS) or medical facilities with minimal equipment. SI was suggested as an accurate tool for identifying early hypovolemic shock, severity of illness, and prognosis, especially in vascular injuries, hemorrhagic events, or trauma patients [20,21,22,23,24,25]. SI was also found to be an independent risk factor and a fast guide for the need of massive transfusion after trauma [25,26,27], and increased blood transfusion is regarded as an independent risk factor correlating with poor prognosis in trauma patients because of the alteration of cytokine levels and inflammatory processes caused by the transfused blood products [20,28]. Jonas et al. concluded that an SI of greater than 0.9 predicts the necessity of intervention for hemostasis with high specificity (93.6%), and by lowering the threshold to ≥0.8, sensitivity is increased to 76.1% [22]. Nakasone et al. demonstrated that SI correlates with extravasation during gastrointestinal hemorrhage via multivariate logistic regression analysis [29]. Kuo et al. reported similar results in patients with pelvic fracture, whereupon relative hypotension is an indicator for TAE regardless of negative contrast extravasation [30]. In our study, there was a significant difference between the TAE group and non-TAE group in the initial SI at presentation, with a mean value of 1.14 and 0.74, respectively (*p* < 0.05). According to the ROC curve, the cut-off value of SI in predicting the necessity of TAE was 0.95 with AUC of 0.8 (Figure 4). Despite modest sensitivity (74%), the high positive predictive value (100%) and specificity (100%) warrant further evaluation or intervention. With a predictor for the necessity of TAE, physicians could provide timely intervention to prevent possible mortality and morbidity in the event of ONB. In our study, 15 patients who had negative findings for angiography had a SI of less than 0.95. Therefore, we would recommend that if a patient is diagnosed with post-traumatic life-threatening ONB with an initial SI of greater than 0.95 following unsuccessful nasal packing, TAE should be initiated as soon as possible to achieve hemostasis, effectively decreasing the amount of blood transfusion required and associated morbidity. We have devised a possible algorithm for the management of life-threatening oronasal bleeding after craniofacial trauma accordingly (Figure 5).

The introduction of damage control resuscitation focuses on hemorrhage control, maintaining hemodynamic stability, and correcting physiological derangements such as coagulopathy, acidosis, and hypothermia to improve patient survival [31]. The development of coagulopathy after trauma has a profound impact on survival and has been identified as an independent predictor of mortality [32,33]. With advancements in the understanding of trauma-induced coagulopathy (TIC) and evolution in resuscitation protocols, there is potential for improvement in prognosis of trauma patients [34]. In trauma patients, post-traumatic hypoperfusion and related tissue injury activate the response cascade of the endothelium, platelets, and the immune system, consequently disrupting the balance of coagulation [35]. Devastating results could occur if such a response is not promptly managed. TIC could be recognized by standard coagulation panels, thromboelastography (TEG), or in combination [33,36]. In this study, the TAE group was found to have significant prolonged INR and thrombocytopenia compared to the non-TAE group. This finding infers that there were more severe tissue injuries in the TAE group, and in turn, TIC. Unfortunately, an effective scoring system for predicting TIC is lacking, and the diverse phenotypes of TIC pose a challenge in making the diagnosis [35,36]. Hence, hemostasis should be the fundamental principal in management, and resuscitation should be tailored individually [37].

In this study, no patients reported a history of congenital coagulopathy, while only one patient was receiving anti-platelet medication due to a history of cerebrovascular accident. During this study, TEG was not widely adopted clinically, so conventional coagulation panels were the only available tools for evaluating coagulopathy. Of the fifty patients included in this study, fifteen patients had prolonged prothrombin time (PT) and thrombocytopenia in various severities. Yet, among these patients, only two patients were clinically recognized as having significant coagulopathy and requiring blood products for correction. As for the patients who met the inclusion criteria but had died from their injuries, TBI was determined to be the cause of death as opposed to coagulopathy. Nevertheless, the association between TIC and TBI has been reported in the literature and the relationship between the two is still evolving [38,39,40]. To this day, TIC remains a challenge in life-threatening hemorrhage, and further studies are warranted for better clinical practice in the context of oronasal bleeding.

In our series, LeFort fractures are associated with greater severity compared to other types of orofacial fracture and account for 42% of all reported fractures. Previous studies have demonstrated that there is a significant correlation between life-threatening ONB and LeFort II or III fractures [1,4]. While Shimoyama et al. reported five patients with LeFort II or III fractures experiencing ONB who had undergone TAE [5], in our study, there was no statistical difference between fracture patterns to recommend assessing the necessity of TAE based on fracture type. The most common bleeder identified in life-threatening ONB is the internal maxillary artery and its branches at the Woodruff plexus. Local compression is rarely effective in this area due to difficulty of access and failure in hematoma formation. Anterior and posterior nasal packing is required to provide adequate tamponade effect. Shimoyama et al. described five patients who achieved hemostasis and temporary reduction by nasal packing after a mid-facial fracture with massive oral bleeding [5]. Soyka et al. reported that successful hemostasis in epistatic patients with a posterior source of nasal bleeding could be achieved by nasal packing in 62% of cases [41], whereas Cogbill et al. found that primary hemostasis with nasal packing proved effective in only 29% of cases [3]. In our study, nasal packing provided successful hemostasis in 22% of patients. Limited effectiveness in nasal packing may be due to the compromise in the integrity of the nasopharyngeal wall secondary to comminuted fractures or multiple bleeders [10]. Though the rate of efficacious hemostasis is suboptimal, nasal packing could be performed with readily available equipment and relatively low risks. This would allow makeshift stabilization of the patient during transferal, which is valuable in trauma management.

Rebleeding after seemingly successful hemostasis following TAE in life-threatening ONB has not been thoroughly discussed. Lee et al. reported that more severe injury, multiple contrast media extravasations during angiography, incomplete TAE, and elevated HR were associated with rebleeding after TAE in blunt liver injury [42]. Chen et al. found that rich collaterals to the bleeding site and a distance of greater than 5 cm between the catheter tip and the bleeding point were significantly related to rebleeding after TAE treatment in the setting of gastrointestinal bleeding [43]. There were seven patients who experienced rebleeding events in this study (Table 2). Six of the seven patients experienced rebleeding within 3 days, while rebleeding was found in the last patient 12 days after initial TAE. Three patients underwent secondary intervention to stop the bleeding. No major consequences or mortality was noted. Application of gelatin foam for embolization tends to have fewer rebleeding events compared to other materials (*p* = 0.05). Gelatin foam has been extensively used in TAE for temporary obstruction, allowing distal embolization by small-size particles. Thus, it is effective in bleeders with rich collaterals. Despite infection or ischemia due to gaseous emboli or erroneous embolization of unintended vessels [44,45], there were no TAE-related complications caused by gelatin foam application in this study.

Dubel et al. reviewed the complication rate for treating epistaxis with TAE, ranging from 0% to 11% with an average major complication rate of 2.5% [46]. With adequate and timely hemostasis, ONB is unlikely to instigate undesirable consequences. In this study, there were no ONB-related mortalities or TAE-related complications. Caution should still be taken for possible complications, including possible iatrogenic cerebrovascular accidents, soft tissue necrosis, ischemic pain, and facial nerve injury.

There are some limitations to the current study. First, the study was conducted in a retrospective manner with a relatively small sample size, resulting in recall bias and a decrease of the statistical power. Second, there were limited cases with penetrating injuries included in this study, suggesting that the result might not apply to patients with similar trauma mechanisms. There was only one patient with penetrating injuries included in this study. Further studies may be warranted to better justify the application of SI in the assessment of patients presenting with penetrating injuries and the management of life-threatening traumatic ONB in such instances. Finally, the lack of consensus in the literature on the definition of post-traumatic life-threatening ONB results in difficulties during the application of our findings in comparative circumstances.

## 5. Conclusions

Early recognition and well-timed intervention are crucial in the management of post-traumatic life-threatening ONB. We demonstrated that TAE and both anterior and posterior nasal packing can be effective treatments for life-threatening ONB among patients with severe craniofacial trauma. Patients initially presenting with SI > 0.95 were more likely to receive TAE and required a significantly greater number of packed red blood cells transfused. Hence, for patients presenting with life-threatening traumatic ONB and a SI > 0.95, TAE should be considered after failed preliminary attempts at nasal packing. When an appropriate, timely intervention is applied, the outcomes for patients who receive angiographic hemostasis are not inferior to those of less severe patients who achieve hemostasis without embolization. Still, further research is needed before a more definitive relationship can be established.

## Figures and Tables

**Table 1:** Demographics of patients with post-traumatic life-threatening oronasal bleeding between angiographic hemostasis and non-angiographic hemostasis groups.

Variables	TAE (*n* = 39)	Non-TAE (*n =* 11)	*p* Value
Age	34.48 ± 19.41	38.54 ± 10.86	0.12
Mechanism			
*MVA*	18 (46.2%)	8 (72.7%)	0.17
*Pedestrian*	8 (20.5%)	0	-
*Fall*	7 (17.9%)	2 (18.2%)	-
*Miscellaneous*	6 (15.4%)	1 (9.1%)	-
Other Injury			
*CNS*	23 (58.9%)	9 (81.82%)	0.28
*Chest*	15 (38.4%)	5 (45.45%)	0.73
*Abdomen*	6 (15.3%)	0	-
*Pelvis*	5 (12.8%)	0	-
*Extremities*	7 (17.9%)	2 (18.18%)	0.31
Fracture			
*LeFort II + III*	16 (41.0%)	5 (45.4%)	1
*NOE*	2 (5.1%)	2 (18.2%)	0.30
*ZMC*	4 (10.2%)	4 (36.4%)	0.05
*Nasal*	2 (5.1%)	0	-
*Mandible*	1 (2.5%)	1 (9.1%)	0.39
Vitals			
*SBP*	107.12 ± 35.74	136.81 ± 27.53	<0.05 *
*HR*	126.84 ± 25.36	98.18 ± 24.28	<0.05 *
*Initial SI*	1.31 ± 0.49	0.73 ± 0.17	<0.05 *
*SI (after nasal packing)*	1.14 ± 0.37	0.74 ± 0.23	<0.05 *
*SI (after TAE)*	0.9 ± 0.31	-	-
GCS	8.17 ± 4.62	7.63 ± 3.72	0.99
Laboratory Studies			
*Hematocrit* (%)	32.6 ± 8.6	34.8 ± 9	0.23
*INR*	1.84 ± 0.95	1.17 ± 0.14	0.042 *
*Platelet* (×10^3^/μL)	162.4 ± 66.9	213 ± 53.9	0.037 *
Blood Transfusion			
*pRBC*	6.6 ± 5	2.9 ± 3.2	<0.05 *
*WB*	1.4 ± 2.9	0.9 ± 2.1	0.77
*FFP*	3.1 ± 4.3	1.5 ± 2.6	0.2
ISS	28.72 ± 10.71	24.54 ± 11.30	0.32
LOS	6.61 ± 5.00	2.54 ± 3.35	1

MVA = motor vehicle accident; CNS = central nerve system; NOE = nasoorbitoethmoidal; ZMC = zygomaticomaxillary complex; SBP = systolic blood pressure; HR = heart rate; SI = shock index; GCS = Glasgow coma scale; pRBC = packet red blood cell; WB = whole blood; FFP = fresh frozen plasma; ISS = Injury Severity Score; LOS = Length of hospital stay. * = significant difference.

**Table 2:** Demographics of patients with traumatic life-threatening oronasal bleeding (ONB) requiring TAE with rebleeding and without rebleeding.

Variables	Rebleeding (*n* = 7)	No Rebleeding (*n* = 32)	*p* Value
Fracture			
*LeFort II + III*	3	13	0.08
*NOE*	0	2	-
*ZMC*	1	3	0.56
*Nasal*	0	2	-
*Mandible*	0	1	-
Material			
*Coils*	1	4	0.96
*Gelfoam*	3	26	0.05
*NBCA*	2	3	0.21
*PVA*	0	2	-
Bleeder			
*IMA*	7	24	0.07
*Facial*	0	5	-
*ECA*	0	3	-
*Lingual*	0	2	-
*Sphenopalatine*	0	1	-
*Ascending pharyngeal*	0	1	-
TAE			
*Extravasation*	6	23	0.56
*Pseudoaneurysm*	5	11	0.1
SI	1.38 ± 0.72	1.30 ± 0.43	0.98
ISS	26.28 ± 11.28	29.38 ± 10.69	0.52
LOS	30.2 ± 12.37	21.96 ± 15.72	0.1

NBCA = N-butyl cyanoacrylate; PVA = polyvinyl alcohol; IMA = internal mammary artery; ECA = external carotid artery; TAE = transarterial embolization; SI = shock index; ISS = injury severity score; LOS = length of hospital stay.

## Data Availability

The data presented in this study are available on request from the corresponding author. The data are not publicly available due to patient privacy.
